# An extended hierarchical ordered probit model robust to heteroskedastic vignette perceptions with an application to functional limitation assessment

**DOI:** 10.1371/journal.pone.0248805

**Published:** 2021-03-25

**Authors:** Zhiyong Huang, Haoxian Wang, Wenyuan Zheng

**Affiliations:** Southwestern University of Finance and Economics, Chengdu, China; Indiana University School of Medicine, UNITED STATES

## Abstract

To improve interpersonal comparability of self-reported measures, anchoring vignettes are increasingly collected in surveys and modeled as the hierarchical ordered probit (HOPIT) model. This paper—based on the idea of psychological distance—relaxes the assumption of vignette equivalence in the HOPIT by allowing for heteroscedasticity in respondents’ perceptions of vignettes. Particularly, we assume that respondents who are more similar to a vignette are more familiar with the condition described and therefore are capable of forming a more precise perception of the vignette. We show evidence in favor of this extended HOPIT through Monte Carlo simulations and an application concerning self-reported vision difficulty from the WHO Study on Global Aging and Adult Health (SAGE).

## Introduction

**Fig 1:**
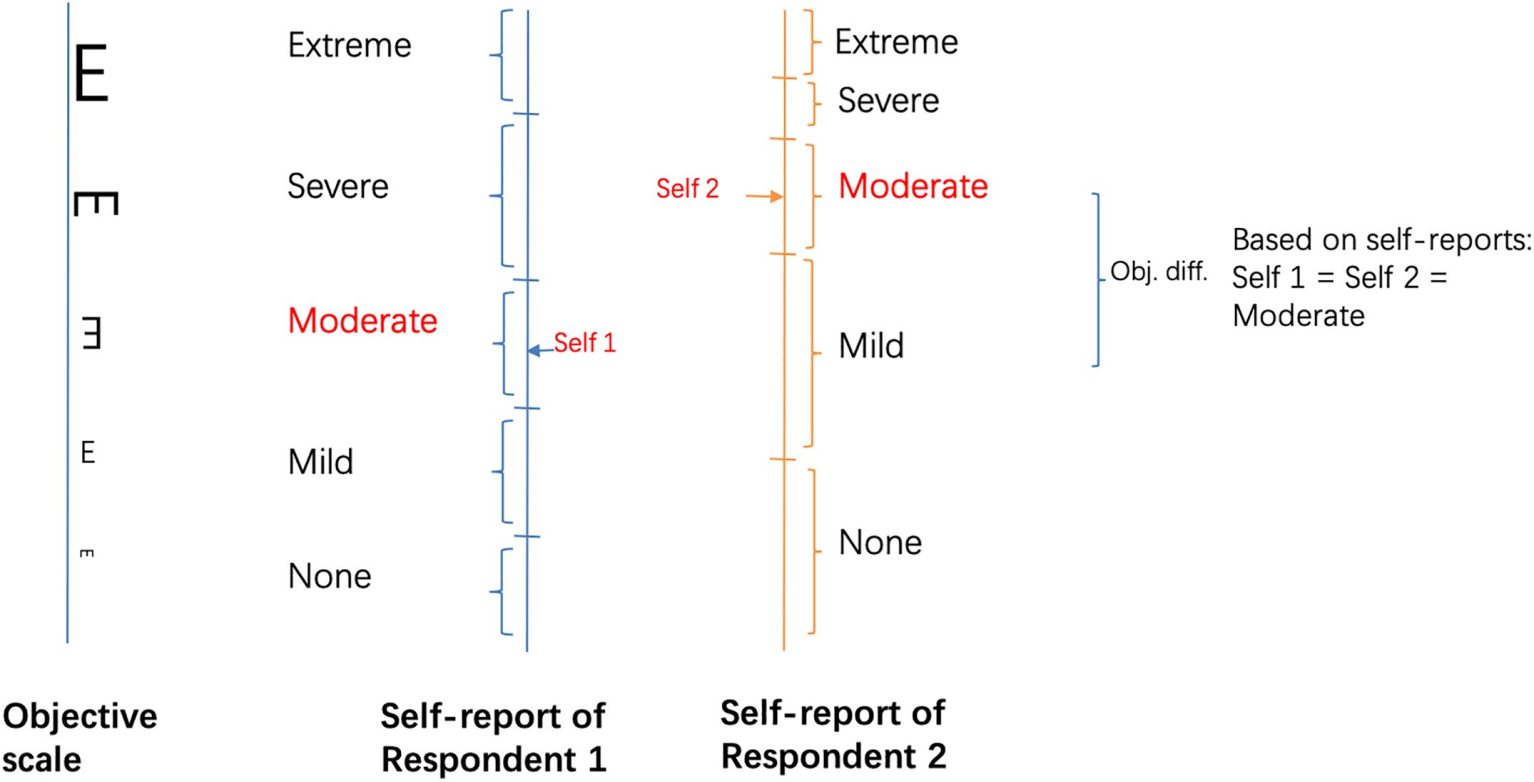
DIF of the item: Self-reported vision. The graph illustrates possible mappings from actual visual acuity to respondents’ self-assessments. Despite the better visual acuity of Respondent 2 than Respondent 1, their self-assessments can be the same when different response scales are used by different respondents. Estimates based on such self-assessments are biased.

Many studies from different domains such as population health or political science are commonly using self-assessments as an alternative to objective measures, which might be infeasible or too costly to collect in surveys. Despite the widespread use of self-assessments, there is some concern on their comparability among individuals with different traits, such as age, gender, socioeconomic status, culture, and nationality [1–3]. While a respondent’s underlying objective condition—which is often the variable of interest—potentially depends on these traits, the response scales underlying her self-assessment may depend on the same traits as well. The use of different response scales in self-assessments by different individuals can compromise the interpersonal comparability of such self-assessed measures. The lack of interpersonal comparability of self-assessments in social surveys has been referred to as differential item functioning (DIF) [1, 4], reporting heterogeneity [5, 6], or cut-point shifts [7]. Fig 1 portrays an example of DIF regarding visual acuity, which is commonly measured by self-report in general surveys. As different reporting scales are adopted, the difference of self-assessments fails to reflect the true difference of vision conditions between the two respondents.

The method of anchoring vignettes proposed by [1] is widely used to adjust for potential heterogeneity in response scales. An anchoring vignette is a brief description of a hypothetical person or situation for a concept relevant to the research question. For instance, one of the anchoring vignettes included in the WHO Study on Global Aging and Adult Health (SAGE) in the domain of visual acuity reads as: “[Eddy] needs a magnifying glass to read small print and look at details on pictures. He also takes a while to recognize objects if they are too far from him. Overall in the last 30 days, how much difficulty do you think [Eddy] had in seeing and recognizing a person he knows across the road (from a distance of about 20 meters)?”

**Fig 2:**
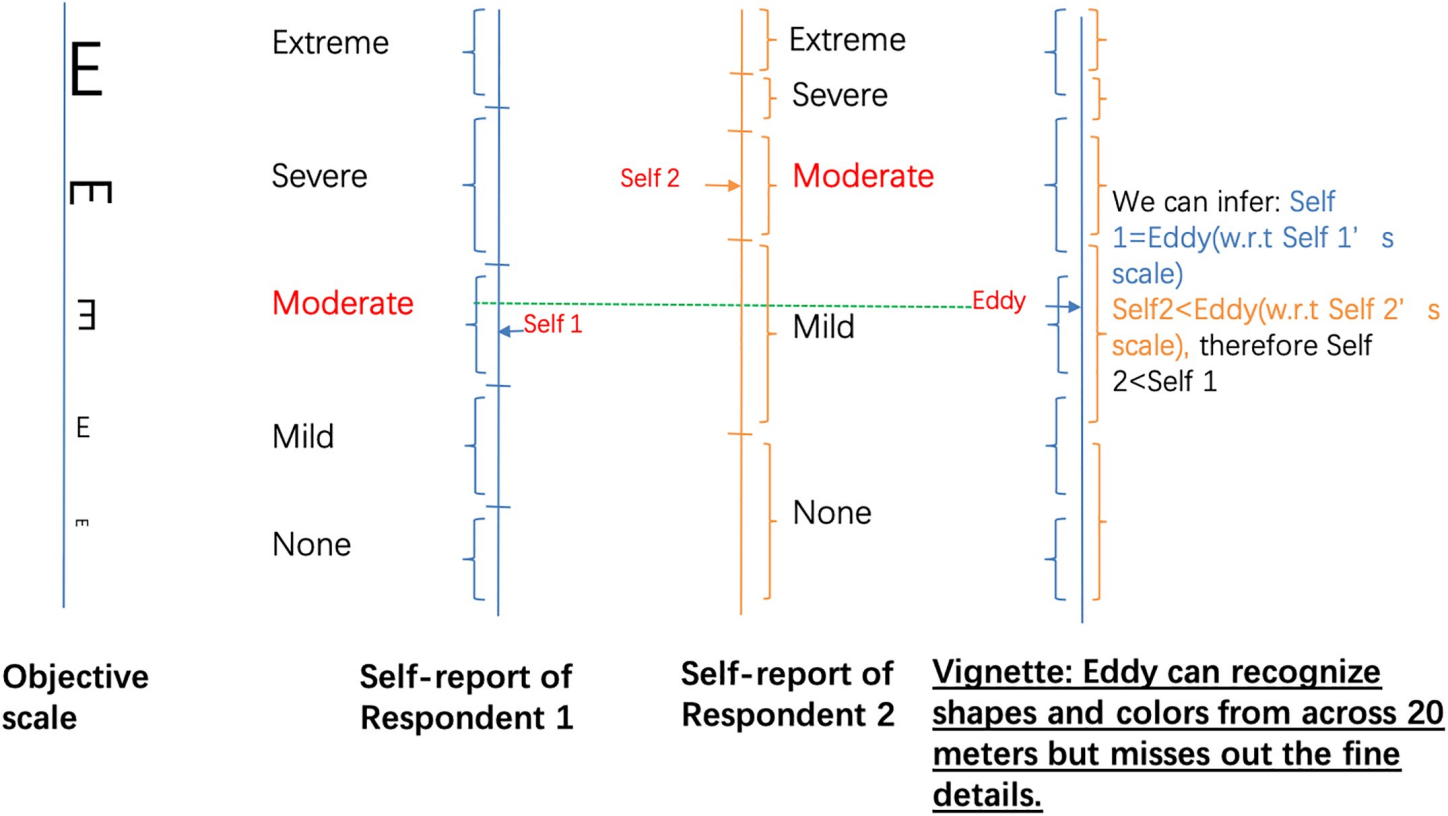
Correction for self-reports using anchoring vignettes.

Respondents are asked to evaluate anchoring vignettes in addition to their self-assessments. Since the objective situation described in a vignette is the same across respondents, responses to vignette questions can help to reveal response heterogeneity and be used to adjust for individual response scales. Once response heterogeneity has been accounted for, true differences between individuals can be identified from their self-assessments. The general idea can be illustrated in Fig 2, where each respondent rates his/her own condition as in Fig 1, but also the condition of a vignette. Using information revealed by anchoring vignettes, individual response scales can be aligned. Self-assessments adjusted by this aligned response scale become comparable.

Anchoring vignettes have been included in many social surveys including the Health and Retirement Study (HRS), the Survey of Health, Aging and Retirement in Europe (SHARE), the WHO Study on Global Aging and Adult Health (SAGE) and the China Health and Retirement Longitudinal Study (CHARLS) to name but a new. The methodology of anchoring vignettes, has been used for a broad range of interpersonal comparisons with regard to health [3, 8–14], healthcare [15, 16], political efficacy [1, 17, 18], life satisfaction [19–22], job satisfaction [23], working disability [2], social status [24], poverty [22] and quality of life [25].

A commonly used parametric model for incorporating anchoring vignettes is the so-called hierarchical ordered probit (HOPIT) model [1, 10, 21, 26]. The validity of the HOPIT model hinges on two assumptions: vignette equivalence and response consistency. Response consistency assumes that the same reporting scale is used by a respondent when evaluating one’s own conditions and anchoring vignettes, while vignette equivalence posits that the perceptions of vignettes are systematically invariant across all respondents.

Vignette equivalence assumes that the perception errors of vignettes are homoskedastic across individuals. This homoskedasticity assumption, however, may be too restrictive. Given several vignettes, a respondent may form a more accurate perception of vignettes that resemble his own condition than those which seem more exotic. Therefore, a vignette can be better perceived by respondents who are similar to the description of the vignette than those who are not familiar with the described condition. For instance, the situation of “Eddy”- the vignette above—might be easily understood and evaluated by an older person with similar vision loss, while a 20-year-old with 20/20 vision may find it much harder to evaluate Eddy’s condition. Even though the 20-year-old can assert that Eddy’s vision is worse than his own, he might still have some difficulty in assessing the degree of severity of Eddy’s visual impairment. As a result, his perception of Eddy’s vision limitation may have a relatively large variance, compared to a person in a more similar situation to Eddy. The post-survey interview in a recent validation study of anchoring vignettes by [27], revealed that some young respondents indeed reported difficulties imaging some vignette scenarios related to limitations such as walking difficulties or chronic pain. The potentially large variance due to the perception distance between one’s condition and vignette scenario may as well contribute to commonly observed ties and inconsistencies in assessments of multiple vignettes, as highlighted by [17] on their discussion on tied or inconsistent vignette rankings:

“… [W]e might reasonably expect respondents to be more likely to give some tied or inconsistent answers among vignettes that are far from their self-assessment even when they correctly rank the vignettes that matter near their value. For example, if we are measuring height and a respondent knew his or her height to within an inch, he or she still might have difficulty correctly ranking the heights of two trees 200 and 206 feet tall, swaying in the breeze. Yet, the same respondent would presumably have no difficulty understanding that both trees are taller than himself or herself” [17, p. 51].

In this paper, we relax the assumption of vignette equivalence by allowing for heteroskedasticity in vignette perceptions. Particularly, we consider situations in which the information revealed by a vignette depends on the similarity/dissimilarity between a respondent and the condition described in that vignette. We assume that the variance of a vignette perception is positively related to the distance between the respondent’s condition and the location of the vignette. Our extension of the HOPIT model has the advantage that vignettes are locally weighted by their distances to the respondent in each respondent-vignette pair. The perception of an anchoring vignette, which is further from the condition of a respondent is allowed to have a larger variance, accounting for higher propensities of ties and inconsistency observed in that respondent-vignette pair. Besides, our extended HOPIT model nests the standard HOPIT as a special case, and can thus be tested against the standard HOPIT using a likelihood ratio test.

Our idea behind the introduction of heteroskedastic variance is closely related to the measurement of psychological distance, defined as the the similarity between one’s direct experience of the “here and now” and hypothetical objects which are not yet directly experienced [28]. The construal level theory in social psychology contends that people perceive hypothetical objects in different ways [29]. People comprehend distant objects more abstractly while interpreting nearby objects in a more concrete way. In our extended HOPIT, we relate the distance between a respondent and a hypothetical situation—in our case a vignette—to the variance of perception, which quantifies the degree of “concreteness” or “abstractness” of the vignette to the respondent.

To the best of our knowledge, our proposed model is the first model that incorporates heteroskedastic vignette perceptions into the HOPIT. [30] adopted a HOPIT model where different vignettes can have different variances, but the variance of any particular vignette is still assumed to be the same for all respondents. Our extension, however, allows the information content of any particular vignette to be different across respondents. A vignette can be understood quite accurately by some respondents while being vaguely understood by others. Compared with the model taken by [30], our extended HOPIT model admits a different variance of vignette perceptions across both respondents and vignettes.

The rest of the paper is organized as follows. Section 2 introduces the extended HOPIT model. Section 3 presents a Monte Carlo study comparing models and an empirical application concerning functional limitation assessment. Section 4 concludes the paper.

## Methods

We model self-assessments and anchoring vignettes jointly. A self-assessment question on vision difficulty, for example, maybe formulated as the following question from SAGE: “In the last 30 days, how much difficulty did you have in seeing and recognizing an object or a person you know across the road (from a distance of about 20 meters)”, with responses as one of, “none”, “mild”, “moderate”, “severe”, or “extreme”. We define anchoring vignettes as descriptions of hypothetical persons in the same domain as self-assessment. A corresponding vignette for the above vision self-assessment reads as “Eddy needs a magnifying glass to read small print and look at details on pictures. He also takes a while to recognize objects if they are too far from him. Overall in the last 30 days, how much difficulty do you think Eddy had in seeing and recognizing a person he knows across the road (from a distance of about 20 meters)”, with responses as one of, “none”, “mild”, “moderate”, “severe”, or “extreme”.

In this section, we first introduce the model specification of the standard HOPIT and then move to our extended model with heteroskedasticity after a discussion of the potential weakness of the standard HOPIT.

### The standard HOPIT model

Let *i*, *i* = 1, …, *N* be respondents, *k*, *k* = 1, …, *K* be response categories, and *j*, *j* = 1, …, *J* be vignette questions. We use *Y* = {*y*_*i*_∣*i* = 1, …, *N*} for the response to the question of self-assessment, *V* = {*V*_*ij*_∣*i* = 1, …, *N*; *j* = 1, …, *J*} for responses to the vignette questions, and *X* = {*x*_*i*_∣*i* = 1, …, *N*} and *Z* = {*z*_*i*_∣*i* = 1, …, *N*} for regressors determining the true status underlying self-assessments and the cut-off points, respectively. Note that *X* and *Z* are not necessarily of the same set, although most empirical studies assume that the two contain the same variables.

We assume that the latent variable of interest takes a linear form

$$\begin{array}{c} y_{i}^{*}=\beta^{\prime }x_{i}+\sigma_{\varepsilon }\varepsilon_{i} \end{array}$$ (1)

where the error term *ε*_*i*_ follows a standard normal distribution, i.e., *ε*_*i*_ ∼ *N*(0, 1). Further, we impose as identification restrictions *β*_0_ = 0 and *σ*_*ε*_ = 1 without loss of generality as in the standard ordered response models.

**Fig 3:**
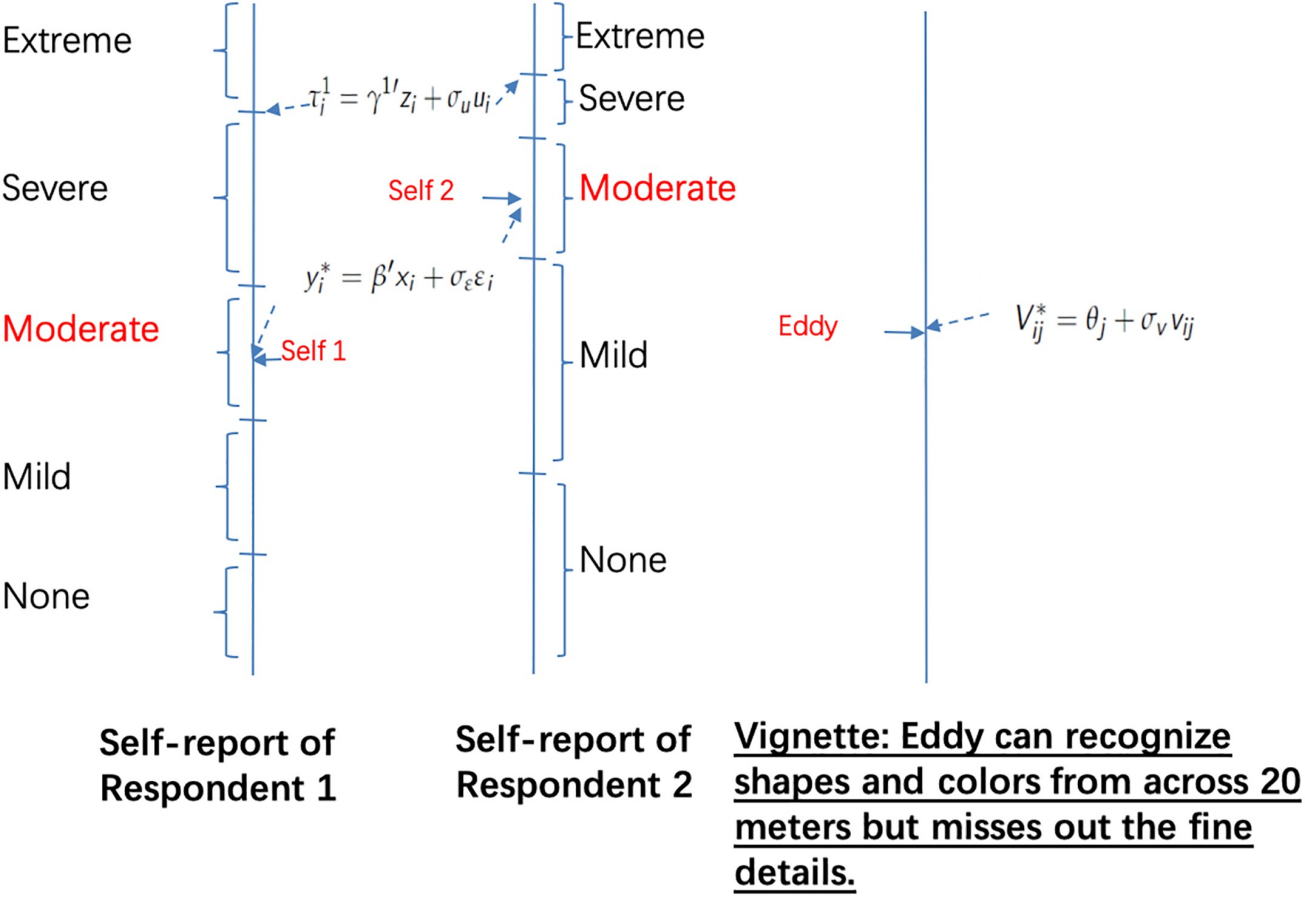
The standard HOPIT model.

The observed categorical responses follows a standard mapping rule given by

$$\begin{array}{c} y_{i}=k\Leftrightarrow \tau_{i}^{k-1}\leq y_{i}^{*}<\tau_{i}^{k} \end{array}$$ (2)

where *τ*_*i*_ denotes individual-specific cut-off points modeled as

$$\begin{array}{c} \tau_{i}^{1}=\gamma^{1}{}^{\prime }z_{i}+\sigma_{u}u_{i}\\ \tau_{i}^{k}=\tau_{i}^{k-1}+\text{exp}(\gamma^{k}{}^{\prime }z_{i}),\;k=2.,\ldots ,K-1\\ \tau_{i}^{0}=-\infty ,\tau_{i}^{K}=\infty \end{array}$$ (3)

where *u*_*i*_ is the unobserved component of cutoff points. The Fig 3 illustrates the model specification of the standard HOPIT model. This model setup is used, for example, by [2, 13, 15, 19, 21].

Given the above setup, the probability that a respondent *i* has a response *k*, conditional on both *u*_*i*_ and *ε*_*i*_, is

$$\begin{array}{ccc} \text{Pr}(y_{i}=k|x_{i},z_{i},u_{i},\varepsilon_{i}) & = & 1\{\tau_{i}^{k-1}\leq \beta^{\prime }x_{i}+\varepsilon_{i}<\tau_{i}^{k}\} \end{array}$$ (4)

where 1{}is the indicator function. Note that in the standard HOPIT model, we can further derive the conditional probability as $\text{Pr}(y_{i}=k|x_{i},z_{i},u_{i})=\Phi (\tau_{i}^{k}-\beta^{\prime }x_{i})-\Phi (\tau_{i}^{k-1}-\beta^{\prime }x_{i})$ by integrating *ε*_*i*_ out. In our extended HOPIT model, however, we need the probability to be conditional on *ε*_*i*_ as well, since *ε*_*i*_ is also incorporated in functions of vignette assessments. In the likelihood function, *ε*_*i*_ will be integrated out as an unobserved individual effect.

Anchoring vignettes are used to identify the effect of the regressor on the outcome from reporting styles. The assumptions needed to ensure the validity of anchoring vignettes are vignette equivalence and response consistency. The assumption of response consistency requires that respondents use the same response scales for both their self-assessments and their vignette ratings.

$$\begin{array}{c} V_{ij}=k\Leftrightarrow \tau_{i}^{k-1}\leq V_{ij}^{*}<\tau_{i}^{k} \end{array}$$ (5)

The assumption of vignette equivalence requires that respondents interpret vignettes in the same way subject to an idiosyncratic error term. Under this assumption, a respondent *i*’s perception of vignette *j* is given by

$$\begin{array}{c} V_{ij}^{*}=\theta_{j}+\sigma_{v}v_{ij},\;v_{ij}∼N(0,1) \end{array}$$ (6)

where *θ*_*j*_ is the location of vignette *j* and *v*_*ij*_ is the idiosyncratic error.

### The extended HOPIT model with heteroskedastic vignette perceptions

In the standard HOPIT model, vignette equivalence (Eq 6) implies that the perception errors of vignettes are homoskedastic across individuals. The homoskedasticity assumption, nonetheless, can be too restrictive. Given several vignettes, a respondent may form a more accurate perception of vignettes that resemble his condition than those which seem more exotic. Therefore, a vignette can be better perceived by respondents who are similar to the description of the vignette than those who are not familiar with the described condition.

In this paper, we relax the assumption of vignette equivalence by considering a particular source of heteroskedasticity regarding vignette perception. Specially, we assume that the information that a vignette reveals depends on the similarity/dissimilarity between a respondent and the vignette, i.e., we assume that the precision of vignette perception is negatively related to the distance between the respondent’s own condition and the vignette,

$$\begin{array}{c} V_{ij}^{*}=\theta_{j}+\sigma_{v}\;\text{exp}(\alpha {\left(\beta^{\prime }x_{i}+\varepsilon_{i}-\theta_{j}\right)}^{2})v_{ij},\;v_{ij}∼N(0,1) \end{array}$$ (7)

where the parameter *α* measures the impact of the similarity/dissimilarity between the respondent and the vignette on the level of noise in a specific respondent-vignette-pair. Note that the respondent’s own condition consists of both an observed part *β*′*x*_*i*_ and an unobserved part *ε*_*i*_.

Our specification of heteroskedasticity is based on the theory of psychological distance, which may describe the temporal distance between the present and the future, the spatial distance between different physical locations, social distance difference between yourself and others, or hypothetical distance between imaging and experienced events [29]. The theory of psychological distance assumes that people think more concretely when faced with an object or an event of a shorter psychological distance while thinking more abstractly on a distant object or event. Whether the hypothetical event transcends into our mindset in a more concrete or a more abstract way would, in turn, affect our perception precision to the event. For example, [31] have found that a concrete mindset often achieves higher accuracy on the estimate of risk events.

When respondents assess anchoring vignettes which are a set of hypothetical events which are not directly experienced by respondents, their perceptions are apt to be affected by the hypothetical distance. As the respondent shows a higher degree of psychological proximity to the vignette, the perception can become more concrete and thus more precise. In our specification, the degree of concreteness (or abstractness) in vignette perception is modeled through the variance in the vignette perception function.

**Fig 4:**
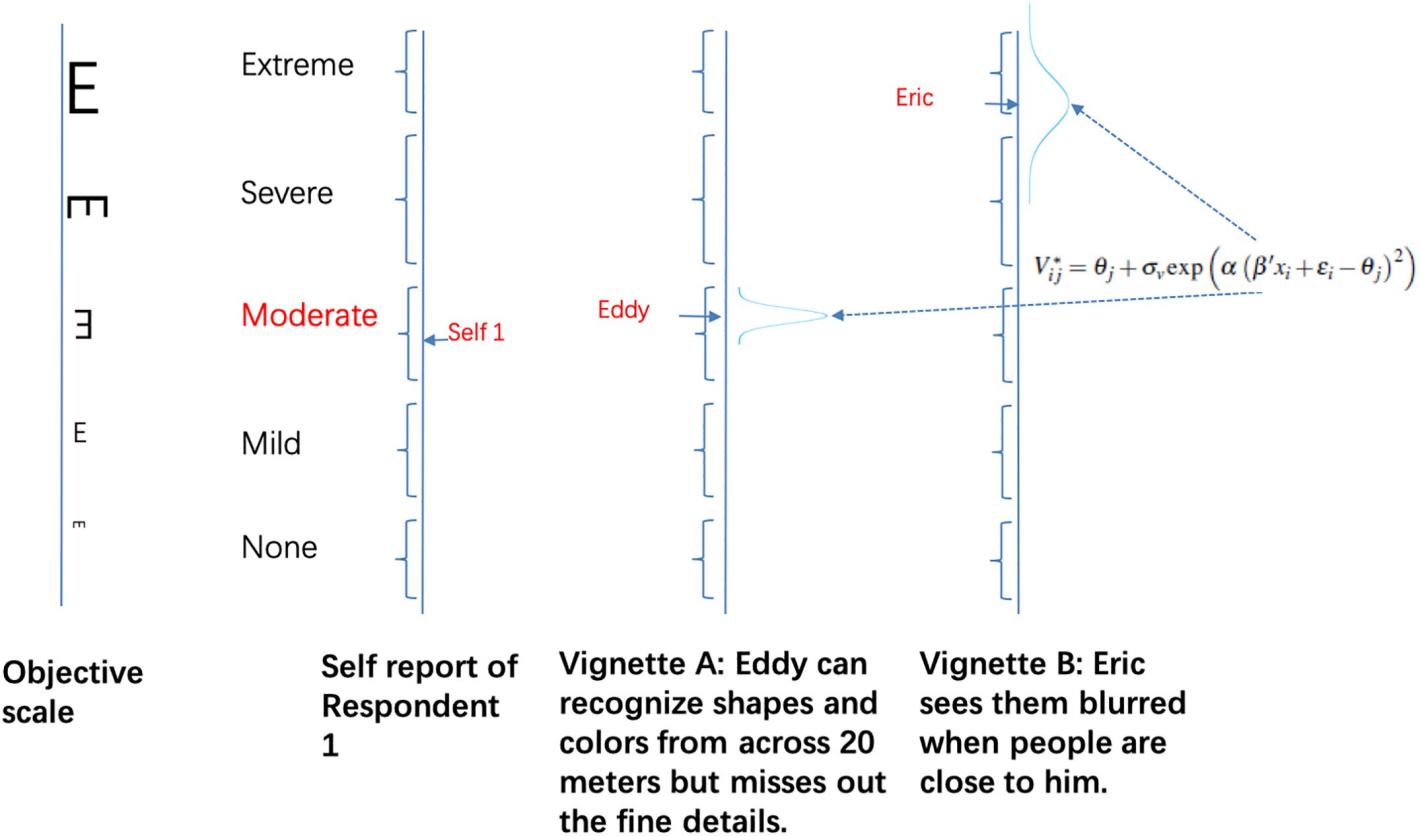
The extended HOPIT with heteroskedastic vignette perceptions.

Under the assumption of heteroskedastic perception, each respondent-vignette-pair is locally weighted by the distance between the respondent’s situation and the vignette. The more similar a vignette is to a respondent, the more precise is the respondent’s perception of the vignette, and the higher the weight the vignette gets in the correction of reporting heterogeneity across respondents. Fig 4 shows one example of heteroskedastic perceptions. A respondent assesses his situation as well as situations of two vignettes, “Eddy” and “Eric”. Eddy’s situation is thereby more similar to the respondent’s condition than the situation of Eric. Following our idea of heteroskedastic vignette perceptions, the respondent would have a more accurate perception of Eddy’s situation than the situation of Eric, as the former is more similar to his condition.

Moreover, when *α* = 0 takes the value of zero, the extended HOPIT model transforms into the standard HOPIT model. Therefore, we can treat the extended HOPIT model as the unrestricted model and the standard HOPIT model as the restricted model, and therefore use LR test to test the extended HOPIT model against the standard HOPIT model. Specifically, the LR statistics can be calculated as -2[log(likelihood of the extended HOPIT model)—log(likelihood of the standard HOPIT model), where the likelihood of the extended HOPIT is obtained from the maximum likelihood estimation of the extended HOPIT model and the likelihood of the standard HOPIT is obtained from the maximum likelihood estimation of the standard HOPIT model.

Under the assumption of response consistency (Eq 5) and our relaxed assumption of vignette equivalence (Eq 7), the probability that a respondent *i* rates a vignette *j* as *k*, conditional on both *u*_*i*_ and *ε*_*i*_, is given by

$$\begin{array}{c} \text{Pr}(V_{ij}=k|x_{i},z_{i},u_{i},u_{i},\varepsilon_{i})\\ =\Phi (\frac{\tau_{i}^{k}-\theta_{j}}{\sigma_{v}\;\text{exp}(\alpha {\left(\beta^{\prime }x_{i}+\varepsilon_{i}-\theta_{j}\right)}^{2})})-\Phi (\frac{\tau_{i}^{k-1}-\theta_{j}}{\sigma_{v}\;\text{exp}(\alpha {\left(\beta^{\prime }x_{i}+\varepsilon_{i}-\theta_{j}\right)}^{2})}) \end{array}$$ (8)

Estimates for the model parameters can be obtained by maximizing the log-likelihood $L=\sum_{i=1}^{N}\text{log}\;\ell_{i}$, with *ℓ*_*i*_ given by

$$\begin{array}{c} \ell_{i}(\beta ,\gamma^{1},\ldots ,\gamma^{K-1},\theta_{1},\ldots ,\theta_{J},\sigma_{v},\sigma_{u},\alpha )=\int \int \prod_{k_{0}=1}^{K}{\left[1\{\tau_{i}^{k_{0}-1}\leq \beta^{\prime }x_{i}+\varepsilon_{i}<\tau_{i}^{k_{0}}\}\right]}^{1\{y_{i}=k_{0}\}}\\ \prod_{j=1}^{J}\prod_{k_{j}=1}^{K}{\left[\Phi (\frac{\tau_{i}^{k_{j}}-\theta_{j}}{\sigma_{v}\;\text{exp}(\alpha {\left(\beta^{\prime }x_{i}+\varepsilon_{i}-\theta_{j}\right)}^{2})})-\Phi (\frac{\tau_{i}^{k_{j}-1}-\theta_{j}}{\sigma_{v}\;\text{exp}(\alpha {\left(\beta^{\prime }x_{i}+\varepsilon_{i}-\theta_{j}\right)}^{2})})\right]}^{1\{V_{ij}=k_{j}\}}\\ d\Phi (\varepsilon_{i})d\Phi (u_{i}) \end{array}$$ (9)

where Φ() is the standard normal cumulative distribution function.

Comparing the likelihood of the extended HOPIT model with that of the standard HOPIT model, one can see that in the extended model the contribution of the perception of a vignette *j* by an individual *i* to the likelihood is weighted by its exponential distance from *i* to *j*.

**Algorithm 1**: Maximum likelihood estimation (MLE) of model parameters

1. Initialization of estimates of model parameters;

2. **while** *estimates of parameters not converging* **do**

 2.1 Evaluate likelihood

  2.1.1 Do importance sampling;

  2.1.2 Do numerical integration using quasi-Monte Carlo method;

 2.2 Update estimates of parameters using interior point approach;

**end**

3. Return estimates of parameters

Estimates of parameters are obtained by the method of maximum likelihood estimation (MLE), which is detailed in Algorithm 1. We use a quasi-Monte Carlo method to evaluate the integral in the likelihood function. In contrast to Monte Carlo methods which draw random sequence, the quasi-Monte Carlo method solves numerical integration using quasi-random sequence which often results in a better rate of convergence. In particular, we use a 2-dimensional Halton sequence for *ε*_*i*_ and *u*_*i*_ that omits initial 1000 points and leaps every 100 points generated. Besides, to reduce the variance of the quasi-Monte Carlo integration and thereby obtain more precise estimates of model parameters for a given number of iterations, we use importance sampling to gain a better coverage on the indicator function within the Monte Carlo integral, as detailed in S3 Appendix.

Simulations and estimations are implemented using MATLAB R2017a (The MathWorks, Inc, Natick, Apple Hill Campus, U.S).

## Results

### Simulation studies

#### Data generating process and model fit measures

In this section, we explore the finite sample performance of our extended HOPIT model through a Monte Carlo study. First, we investigate the potential bias of standard HOPIT models, which are misspecified in the presence of perception heteroskedasticity. Second, we examine whether our extended HOPIT models produce comparable results with the standard HOPIT model in the absence of perception heteroskedasticity.

The simulated datasets are generated from the extended HOPIT model as follows:

- Set the number of response categories *K* to be 3, which corresponds to a 3-point Likert scale commonly used in social surveys.
- Generate 10 exogenous variables: variables *x*_*i*1_ − *x*_*i*5_ are drawn from *U*(0, 1) distributions corresponding to continuous regressors such as age or years of schooling, and variables *x*_*i*6_ − *x*_*i*10_ are drawn from Bernoulli distributions representing binary regressors like sex or dumminized categorical variables like country of residence.
- For parameters of coefficients, assign both positive and negative values representing both positive and negative effects, as described in the second column in Table 1.
- Set parameters of the cutoff equations to be the same as the outcome equations so that the reporting heterogeneity is present.
- Set the number of vignettes *J* to be 1, 3 and 5, which agrees with the number of vignettes usually included in surveys such as SAGE.
- Set the parameter of heteroskedasticity *α* to 0, 0.02 and 0.05, calibrated according to the estimation of real data, as shown in the following section.
- Set the number of observations *N* to 1, 000 and 2, 000, which is at the same scale of observations included in surveys such as SAGE.
- Each Monte Carlo experiment is replicated for 100 times.

Our empirical example, which is detailed in the following section, suggests *α* is around 0.03. We, therefore, simulate three data generating processes (DGPs) in line with this scale of *α*: no heteroskedasticity (*α* = 0), weak heteroskedasticity (*α* = 0.02) and strong heteroskedasticity (*α* = 0.05). When heteroskedasticity (weak or strong) is present, the standard HOPIT model—which does not take the heteroskedasticity into account—is under-specified. In contrast, our extended HOPIT model is over-specified when there is no heteroskedasticity.

For each generated dataset, we estimate both the standard HOPIT model and our extended HOPIT model. For each Monte Carlo experiment, multiple measures are used to evaluate model fit. Firstly, we calculate the mean squared error (MSE) of the model parameters across all replications of the experiment. Secondly, the mean values of the log-likelihoods and Akaike information criteria (AICs) are reported to compare the overall model fit. Third, the dependence measured as Pearson’s correlation and Kendall’s tau between the predicted outcome and simulated outcome is calculated. Finally, since the standard HOPIT model is nested in our extended HOPIT model, likelihood ratio tests are also employed to compare the two models. The test results are summarized by the rejection rate of the standard HOPIT model across all replications in each experiment. Note that the size of the likelihood ratio test is 0.05 and the test statistic is compared with the critical value of a standard *χ*^2^-distribution.

#### Results

The results from our simulations depend on the specifications for the value of *α*, the number of vignettes *J* and the number of observations *N*.

Table 1 summarizes the results of experiments when the parameter of heterogeneity *α* = 0.02 and the number of vignettes *J* = 5. In terms of parameter estimates, the extended HOPIT results in more accurate estimates, as measured as smaller MSE of parameter estimates, than the standard HOPIT. In terms of model fit, while the LR test is in favor of the extended HOPIT and the AIC is much smaller for the extended HOPIT, the correlations measured as Pearson’s correlation and Kendall’s tau between predictions and true outcomes show little difference between the standard and extended HOPIT models.

**Table 1:** MSE of parameters and model fit for experiments where *α* = 0.02 and *J* = 5.

Parameter	True value	*N* = 1000		*N* = 2000	
		Standard HOPIT	Extended HOPIT	Standard HOPIT	Extended HOPIT
*β*_1_	1	0.795	0.042	1.184	0.028
*β*_2_	-1	0.848	0.058	1.800	0.026
*β*_3_	1	0.741	0.049	0.867	0.024
*β*_4_	-1	0.512	0.054	1.586	0.027
*β*_5_	1	0.955	0.049	1.664	0.024
*β*_6_	-1	0.886	0.018	1.205	0.015
*β*_7_	1	1.371	0.020	0.937	0.014
*β*_8_	-1	1.070	0.025	0.890	0.015
*β*_9_	1	0.989	0.020	0.735	0.016
*β*_10_		0.844	0.021	0.698	0.015
Goodness of fit					
*AIC*		5600	5227	11111	10367
*Pearson’s ρ*		0.786	0.789	0.789	0.791
*Kendall’s τ*		0.579	0.582	0.583	0.585
*LR test* *(% rej. α* = 0.05*)*		100		100	

Table 2 summarize the results of experiments when the parameter of heterogeneity *α* = 0.05 and the number of vignettes *J* = 5. The extended HOPIT shows a much smaller MSE than the standard model. When we test extended HOPIT against standard HOPIT, the standard model is always rejected. In both experiments, the extended HOPIT model has a smaller AIC than the standard HOPIT model. The correlation measured as Pearson’s correlation and Kendall’s tau between predicted and true outcomes for the extended HOPIT is also much larger than the standard model.

**Table 2:** MSE of parameters and model fit for experiments where *α* = 0.05 and *J* = 5.

Parameter	True value	*N* = 1000		*N* = 2000	
		Standard HOPIT	Extended HOPIT	Standard HOPIT	Extended HOPIT
*β*_1_	1	2.332	0.023	1.003	0.015
*β*_2_	-1	1.806	0.026	2.220	0.014
*β*_3_	1	3.513	0.022	2.665	0.012
*β*_4_	-1	3.080	0.021	1.072	0.009
*β*_5_	1	5.074	0.017	1.830	0.013
*β*_6_	-1	0.675	0.008	0.800	0.007
*β*_7_	1	1.407	0.007	1.238	0.004
*β*_8_	-1	0.812	0.011	0.925	0.006
*β*_9_	1	0.900	0.010	1.237	0.006
*β*_10_	-1	1.467	0.007	0.695	0.007
Goodness of fit					
*AIC*	-	8811	7848	17531	15587
*Pearson’s ρ*	-	0.644	0.790	0.699	0.790
*Kendall’s τ*	-	0.447	0.582	0.495	0.584
*LR test* *(% rej. α* = 0.05*)*	-	100		100	

For experiments when *α* = 0, although the extended HOPIT model is over-specified in this case, we do not observe much information loss using the extended HOPIT model against the standard HOPIT model, which results are included in the S1 Appendix. Firstly, we notice that the estimate of *α* is always near its true value of zero. The bias of the estimate shrinks as the number of observations or the number of vignettes increases. Secondly, the standard HOPIT model and the extended HOPIT model have almost equal log-likelihoods, AICs, and correlations. Lastly, the two models exhibit the same level of unbiasedness implied by the similar MSEs of parameters.

Overall, our Monte Carlo experiments show that, in the presence of heteroskedasticity of vignette perceptions, the extended HOPIT model has a better model fit than the standard HOPIT model in terms of the MSEs of the parameters, the AICs and LR tests, and correlation measures. Moreover, in the absence of heteroskedasticity, no information loss has been found using the extended HOPIT model in general. However, it is worth noting, when sample sizes are very small, say 100 or 200, the extended HOPIT model may have more volatile estimates than the standard HOPIT model, as suggested in an even wider range of experiments (results are available upon request from authors).

### Empirical application

#### Data

As shown in the simulation study, the advantage of our extended HOPIT model against the standard HOPIT depends on the size of potential heteroskedasticity, the number of observations, and the number of vignettes available in the dataset. In our empirical application, we use data of visual acuity from the WHO Study on Global Aging and Adult Health (SAGE), which is publicly available from the Inter-university Consortium for Political and Social Research (ICPSR) (https://www.icpsr.umich.edu/web/ICPSR/studies/31381). SAGE asked respondents to complete self-assessment visual acuity and questions regarding vignettes. Each self-assessment was supplemented with five vignette questions, which described varying levels of functional limitations. Questions are detailed in Table 3.

**Table 3:** Questions from SAGE on self-assessments and anchoring vignettes on visual acuity.

	Questions
Self-assessment	In the last 30 days, how much difficulty did you have in seeing and recognizing an object or a person you know across the road (from a distance of about 20 meters)?
Vignette 1	[X] can read words in newspaper articles (and can recognize faces on a postcard size photograph). He can recognize shapes and colors from across 20 meters but misses out the fine details. Overall in the last 30 days, how much difficulty do you think [X] had in seeing and recognizing a person he knows across the road (from a distance of about 20 meters)?
Vignette 2	[X] only reads if the text is in very large print, such as 10 lines per page. Otherwise she does not read anything. Even when people are close to her, she sees them blurred. Overall in the last 30 days, how much difficulty do you think [X] had in seeing and recognizing a person he knows across the road (from a distance of about 20 meters)?
Vignette 3	[X] needs a magnifying glass to read small print and look at details on pictures. He also takes a while to recognize objects if they are too far from him. Overall in the last 30 days, how much difficulty do you think [X] had in seeing and recognizing a person he knows across the road (from a distance of about 20 meters)?
Vignette 4	[X] can read words in newspaper articles (and can recognize faces on a postcard size photograph). He can recognize familiar people’s faces all the time and picks out most details in pictures from across 20 meters. Overall in the last 30 days, how much difficulty do you think [X] had in seeing and recognizing a person he knows across the road (from a distance of about 20 meters)?
Vignette 5	[X] cannot detect any movement close to the eyes or even the presence of a light. Overall in the last 30 days, how much difficulty do you think [X] had in seeing and recognizing a person he knows across the road (from a distance of about 20 meters)?

Table 4 shows the distributions of the self-assessed visual difficulty and vignette responses. The majority of the respondents perceive no physical limitations, and only a few have “severe” or “extreme” self-assessments. The distributions of the vignettes differ greatly, indicating that the information content of a vignette, which is the degree of limitation in our example, varies significantly.

**Table 4:** Self-assessment and vignette evaluations (%).

	Self-assessment	Vignette 1	Vignette 2	Vignette 3	Vignette 4	Vignette 5
None	59.81	30.43	5.98	3.91	71.77	3.89
Mild	23.70	36.31	12.99	16.59	12.82	4.54
Moderate	11.60	22.96	29.02	32.92	10.37	7.61
Severe	4.33	9.00	41.37	37.63	4.61	22.00
Extreme	0.56	1.30	10.64	8.93	0.42	61.96

Note: *N* = 7153

In this application, we would examine how the actual health status, as well as the health perception of the respondents, are affected by socio-demographic factors such as age, gender, education level, and, most likely, country of residence. Self-assessed vision difficulty is studied using both the standard HOPIT model and the extended HOPIT model. We keep individuals with no missing values for any self-assessment, vignette, and covariate of interest in our samples. To maintain as much information as possible, we use different samples for the analyses of different domains.

Table 5 describes the covariates in our analysis, including dummy variables for age-groups of ten years, education levels categorized as primary, secondary, and higher levels of education, gender, and country of residence. The summary statistics of these variables are shown in Table 6, where we observe that about half of the respondents are female, most of the respondents only have completed primary or secondary education and more than 38% respondents live in China.

**Table 5:** Description of covariates.

Covariate	Description
Age	We include dummy variables for age groups of ten years, namely, under 50 (baseline group), 50-59, 60-69, 70-79, and 80 or above.
Education	In SAGE, education is measured as (1) less than primary education, (2) the primary school completed, (3) the second school completed, (4) high school completed, (5) college, pre-university, or university completed, and (6) postgraduate degree completed. We define level (1) and (2) as primary education (baseline category), (3) and (4) as secondary education, and (5) and (6) as higher education.
Female	A dummy variable for gender.
Country of residence	We include dummy variables for countries of residence including China, Ghana, India, Mexico, and South Africa.

**Table 6:** Summary statistics.

	Mean	Std. dev.
Under 50 years	0.216	0.411
Age 50-59 years	0.359	0.480
Age 60-69 years	0.246	0.431
Age 70-79 years	0.140	0.347
Over 80 years	0.039	0.193
Female	0.514	0.500
Primary education	0.446	0.497
Secondary education	0.455	0.498
Higher education	0.100	0.299
China	0.385	0.487
Ghana	0.090	0.286
India	0.220	0.414
Mexico	0.076	0.266
Russia	0.138	0.345
South Africa	0.091	0.288
*N*	7153	

#### Results

Table 7 presents parameter estimates (of outcome equations) using both the standard HOPIT model and our extended HOPIT model. The complete estimate of all parameters is provided in the S2 Appendix. In addition, we present estimates of ordered probit model for comparison. We observe that the extended HOPIT has a smaller AIC than the standard HOPIT model and we reject the standard HOPIT model by the likelihood ratio test. This implies that the extended HOPIT has a better model fit than the standard HOPIT model.

**Table 7:** Estimates of self-assessed vision difficulty.

	Ordered probit		Standard HOPIT		Extended HOPIT	
	Estimate	t-statistic	Estimate	t-statistic	Estimate	t-statistic
Age 50-59 years	0.704***	13.982	0.682***	13.512	0.683***	17.931
Age 60-69 years	1.008***	19.124	0.958***	18.094	0.962***	37.019
Age 70-79 years	1.256***	21.448	1.171***	18.757	1.163***	33.642
Over 80 years	1.520***	19.561	1.426***	19.590	1.454***	8.955
Female	0.182***	5.688	0.159***	4.022	0.157***	6.126
Secondary education	-0.165***	-4.592	-0.187***	-4.560	-0.209***	-6.544
Higher education	-0.254***	-4.877	-0.244***	-4.612	-0.232***	-4.847
Ghana	0.714***	12.774	0.458***	8.435	0.364***	6.461
India	0.715***	16.140	0.395***	8.190	0.088**	2.349
Mexico	0.216***	3.662	0.094***	1.668	-0.165***	-6.362
Russia	0.249***	4.785	0.092*	1.841	-0.074***	-2.701
South Africa	0.361***	6.542	0.212***	3.958	0.071	1.324
*σ*_*u*_	-	-	0.332***	31.749	0.312***	15.824
*σ*_*v*_	-	-	0.961***	50.897	0.926***	25.311
*α*	-	-	-	-	0.031***	23.961
AIC	103,485		102,210		101,538	

Note:

* *p* < 0.1,

** *p* < 0.05,

*** *p* < 0.01.

*N* = 2, 250. LR test (the extended HOPITas the full model and standard HOPIT as the reduced one): *χ*^2^ = 574.117, *p* − *value* = 0.000.

Estimates from the standard and extended HOPIT models differ. While estimates of coefficients of age, gender, and education are very similar between models, the standard and extended HOPIT models provide very different estimates of country effects. According to the standard HOPIT model, China (the reference group) has a lower prevalence of visual difficulty than all other countries including Ghana, India, Mexico, Russia, and South Africa. In contrast, the extended HOPIT model predicts that adults in Mexico and Russia are less likely to experience vision difficulty than their Chinese counterparts.

These two models make similar estimates on variables such as sex and age, but rather different estimates on country dummies, suggesting the dominant role of inter-country differences compared to intra-country differences in determining reporting heterogeneity. To see this, we compare estimates of the ordered probit model with both the standard and extended HOPIT models. We can already see that ordered probit and standard HOPIT produce very different estimates on country variables, but not so much on other variables, which indicates that country difference is the primary source of reporting heterogeneity. The extended HOPIT makes a further correction on the reporting heterogeneity by allowing different weights on vignettes.

The estimate of the parameter *α*, which measures the degree of heteroskedasticity, is statistically significant. To assess the degree of heteroskedasticity in the error term of the vignette equation (Eq 7), we evaluate the heteroskedastic error (after integrating out the unobserved *ε*_*i*_) which is given by

$$\begin{array}{c} E_{\varepsilon_{i}}\{\sigma_{v}\;\text{exp}(\alpha {\left(\beta^{\prime }x_{i}+\varepsilon_{i}-\theta_{j}\right)}^{2})\} \end{array}$$

The derivation of the expression is included in the S4 Appendix. We compare this heteroskedastic error with the standard error in the standard HOPIT.

**Fig 5:**
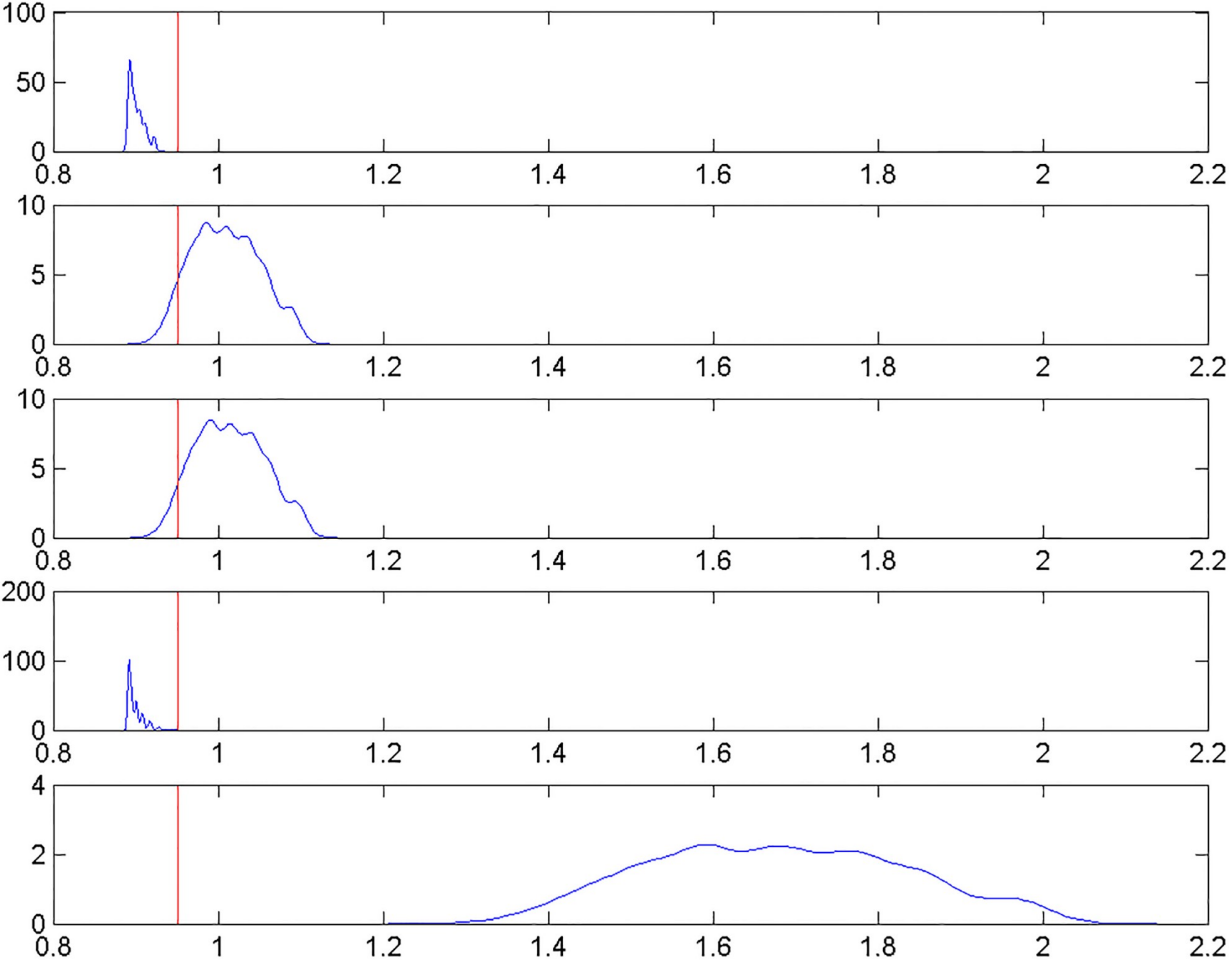
Probability density of heteroskedastic errors with data of vision. Probability densities of heteroskedastic errors of the five vignettes are plotted from top to bottom. The straight red line indicates the standard error from the standard HOPIT model.

Fig 5 shows the probability density of the heteroskedastic errors for each vignette for the analysis of vision. The standard errors are different for each vignette. For most respondents, the first and the fourth vignettes have standard errors smaller than the standard error in the standard HOPIT, while the other three vignettes, especially the last one, have standard errors larger than that in the standard HOPIT. For a given vignette, standard errors also differ across respondents. Especially for the last vignette, standard errors show a relatively large dispersion. Given the extreme scenario described in the last vignette, it is not surprising to observe that on average respondents are less precise in their perception and the degree of precision varies greatly among respondents.

We can safely reject the standard HOPIT based on our test on the significance of *α* which measures the degree of perception heteroskedasticity in our extended model. Yet, the standard HOPIT can also be rejected when other maintained assumptions such as response consistency are violated. If so, our extended HOPIT model could be favored by test results simply because it captures other aspects of model deviation from the truth. If the vignette perception is directly observable, the heteroskedasticity assumption can be easily tested by an auxiliary regression which regresses the squared residuals derived from Eq 7 on the distance between vignettes and respondent health indexes. However, the ordinal nature of vignette assessments mapped from the latent unobservable vignette perceptions precludes such a test. Although an overidentification test like ours could also be affected by model deviation other than vignette perception heteroskedasticity, our extension seems to be able to capture such deviation better than the standard HOPIT indicated by a better overall model fit and hence result in a better estimate of the outcome equation.

## Conclusions

The HOPIT model introduced by [1] has been used in a large number of studies to account for reporting heterogeneity of self-assessments when anchoring vignettes are available. The validity of the model, nevertheless, relies on the assumptions of response consistency and vignette equivalence. The assumption of vignette equivalence assumes that perceptions of vignettes are the same across individuals. In this paper, we relax the assumption of vignette equivalence by allowing for heteroskedasticity of perceptions of vignettes across individuals. Particularly, we assume that the perception precision of a vignette by an individual is negatively proportional to the (exponential) distance between the individual and the vignette, which measures the similarity/dissimilarity between the individual and the vignette.

A series of Monte Carlo simulations show that the extended HOPIT model has a better model fit than the standard HOPIT in terms of MSEs of the parameters, AICs, LR tests, and correlation measures in the presence of heteroskedasticity of vignette perceptions. In the absence of heteroskedasticity, we find almost no information loss using the extended HOPIT model.

When we adopt the extended HOPIT model to analyze self-assessed visual difficulty using data from SAGE, we find that the extended HOPIT model has a better model fit than the standard HOPIT model, as indicated by both information criteria and likelihood ratio tests. Besides, the extended HOPIT provides very different estimates for some parameters compared to the standard HOPIT.

Overall, compared with the standard HOPIT model, our extended HOPIT model facilitates a more flexible way of utilizing information of anchoring vignettes and seems to often have a better model fit than the standard HOPIT model. Our empirical example suggests that the extended HOPIT should be considered for studies with highly heterogeneous samples, especially for between-population surveys, where heteroskedasticity of vignette perceptions is more likely to be present.

One potential disadvantage of our model is that we assume the similarity/dissimilarity constituents the only source of heteroskedasticity of vignette perceptions. One of the future research directions on the extension of the standard HOPIT model would be finding statistical models which allow for a more generic form of heteroskedasticity and meanwhile impose no identification difficulty.

Our extended model also sheds light on the vignette design using the measure of heteroskedasticity in each respondent-vignette pair. The standard HOPIT model, which assumes variance in vignette perception is the same across different individuals. Vignettes which have the smaller variance estimates are deemed to contain more information and therefore selected in subsequent surveys. Yet, application of this criterion may result in dismissing vignettes that are only precisely perceived by a particular group of respondents. Based on the estimates of our extended HOPIT model, we can evaluate the information content of a given vignette for every subgroup of respondents and select vignettes accordingly. For example, if we are particularly interested in the health condition of an SES group, we could calculate the standard error of vignette perception of each vignette regarding this group and select vignettes or design new vignettes with the least perception variance.

## Supporting information

S1 Appendix Simulation results. (TEX)

S2 Appendix Estimates of the standard and extended HOPIT models. (TEX)

S3 Appendix Algorithm used for important sampling of the likelihood function. (TEX)

S4 Appendix Calculation of the heteroskedastic error. (TEX)

S1 File Matlab cods for estimation using the extended HOPIT model. (M)

S2 File Matlab cods for estimation using the standard HOPIT model. (M)

S3 File Matlab cods for estimation using the extended HOPIT model. (M)

S4 File Matlab cods for simulated data generation. (M)

S5 File Matlab code for simulation setup. (M)

S6 File Matlab cods for estimation with simulated data. (M)

S1 Data Matlab cods for empirical application. (CSV)
